# Supplementary figures and images for: Improved birth rates via rehydration of mouse freeze-dried spermatozoa using high-temperature ultrapure water
Source: PLoS One. 2026 Jan 5;21(1):e0333682. doi: 10.1371/journal.pone.0333682 (PMC12768283; doi:10.1371/journal.pone.0333682)

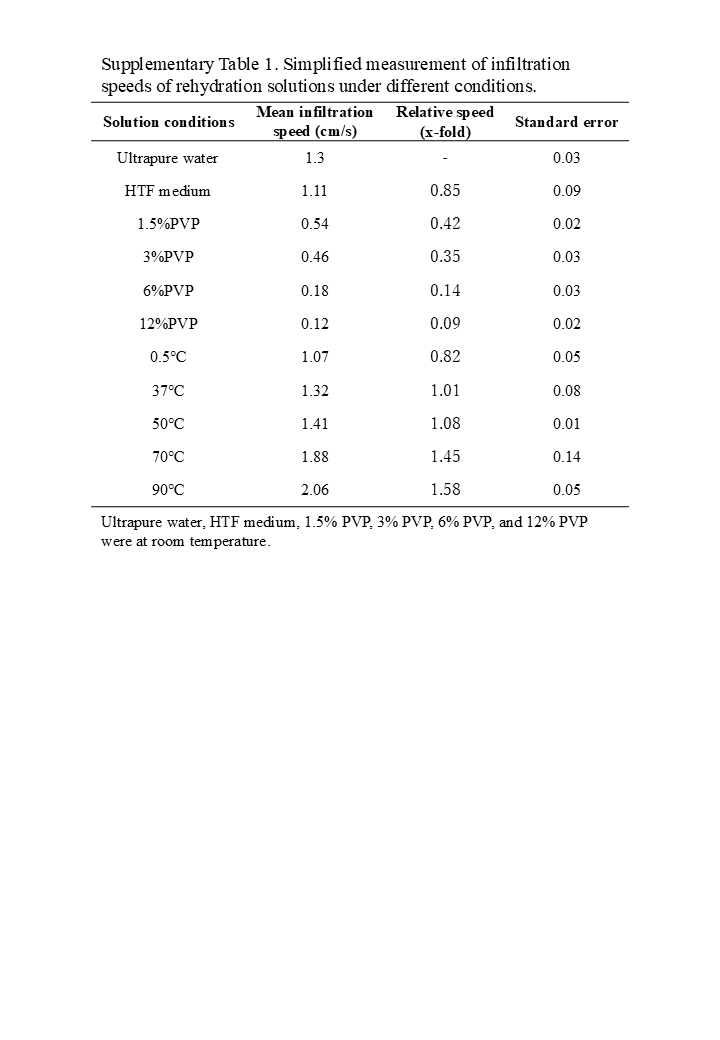

Supplement: S1 Table — (TIF) [file pone.0333682.s001.TIF]

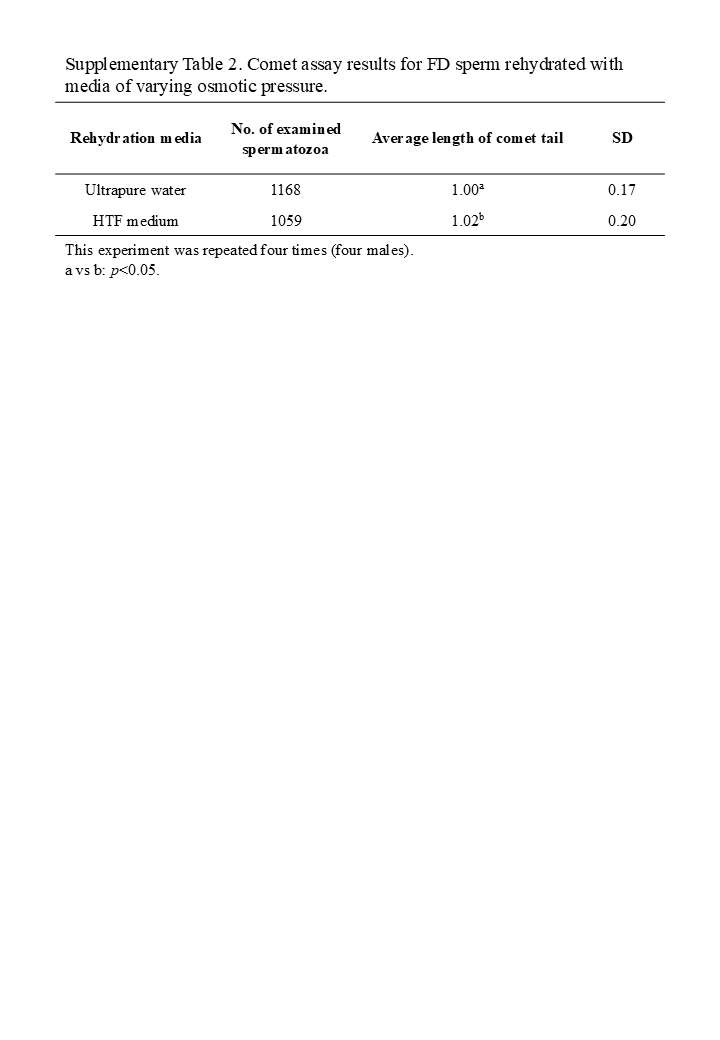

Supplement: S2 Table — (TIF) [file pone.0333682.s002.TIF]

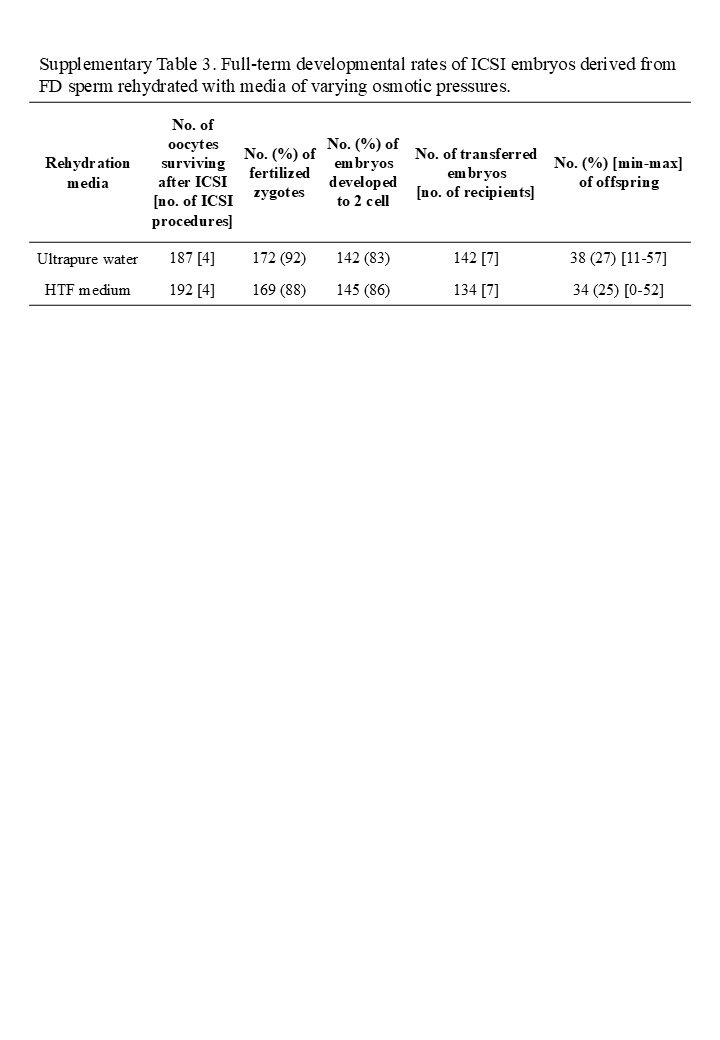

Supplement: S3 Table — (TIF) [file pone.0333682.s003.TIF]

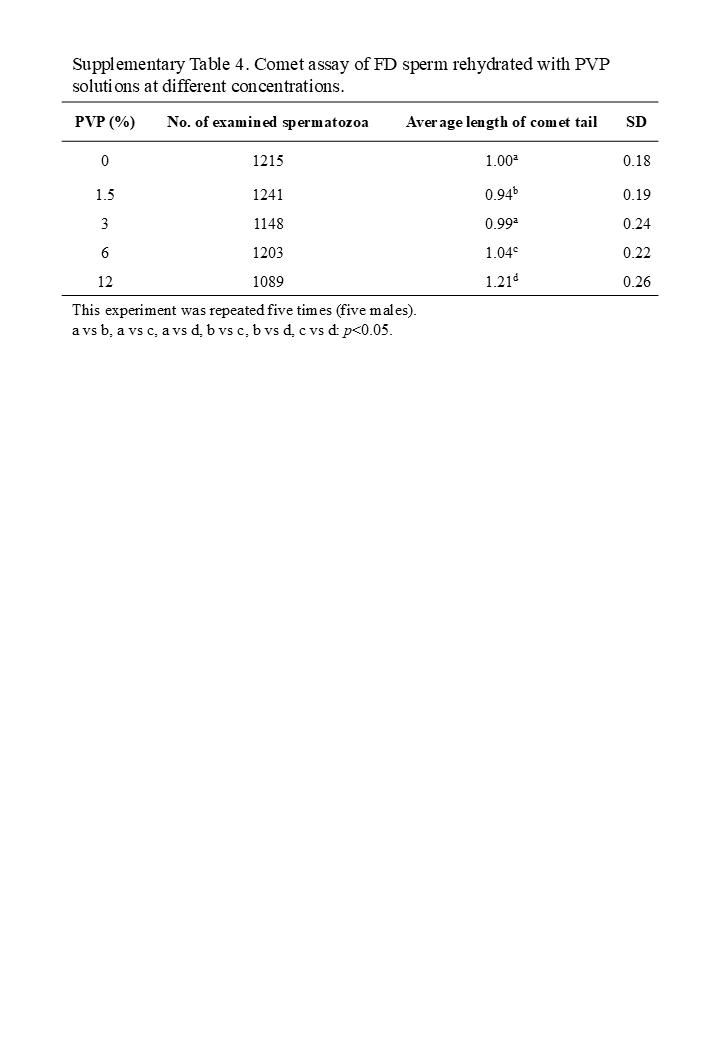

Supplement: S4 Table — (TIF) [file pone.0333682.s004.TIF]

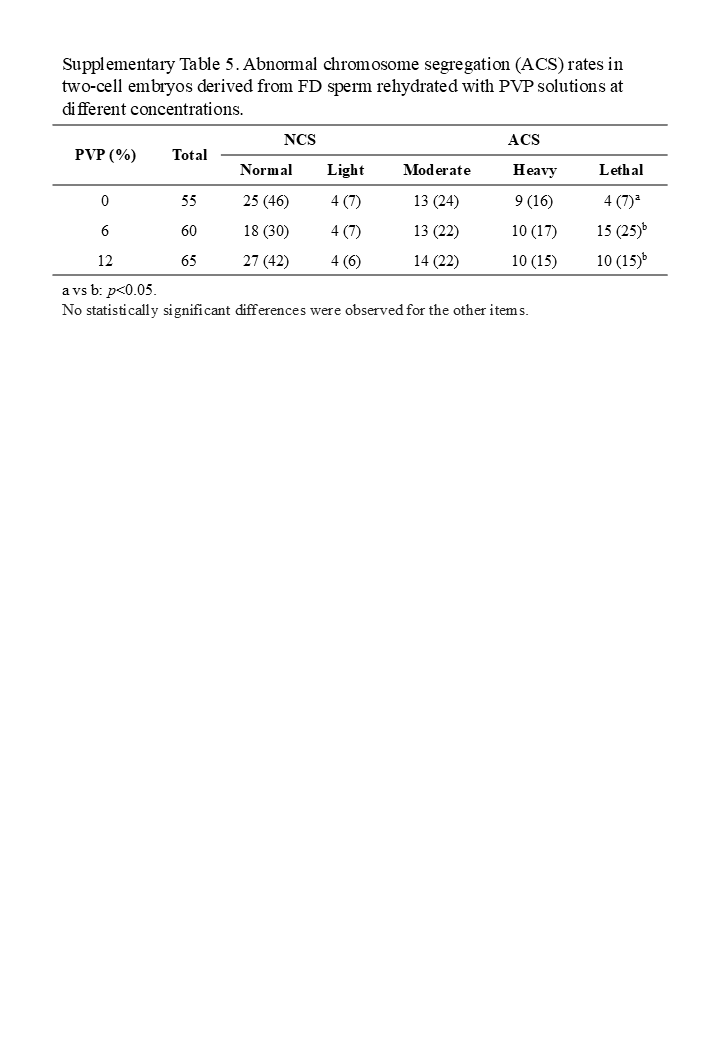

Supplement: S5 Table — (TIF) [file pone.0333682.s005.TIF]

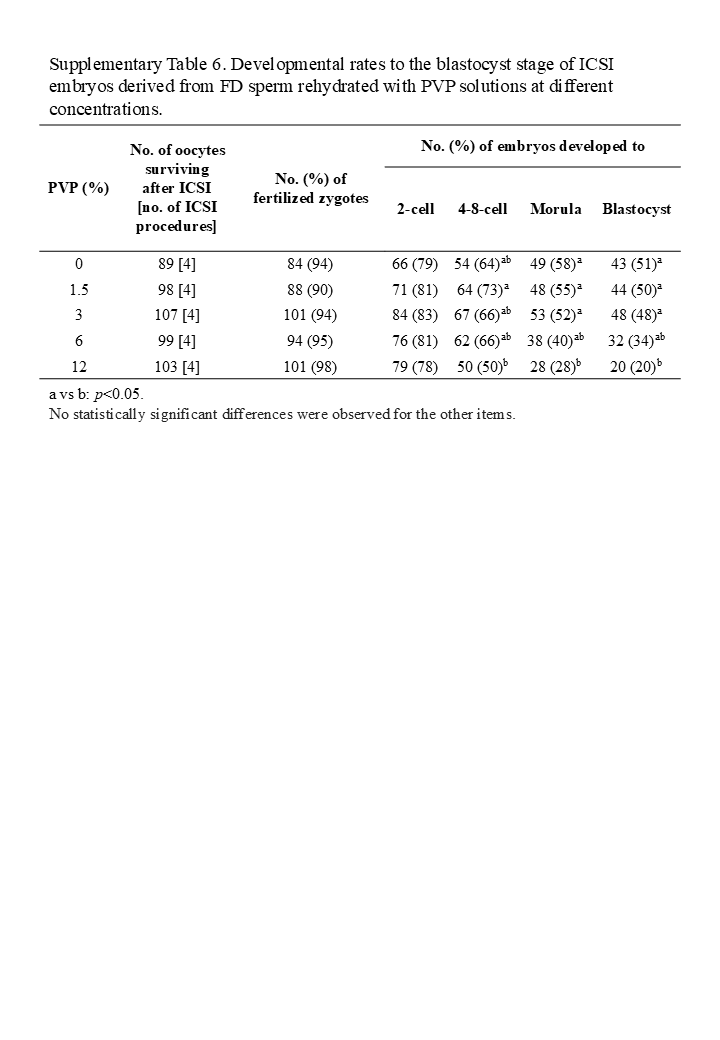

Supplement: S6 Table — (TIF) [file pone.0333682.s006.TIF]

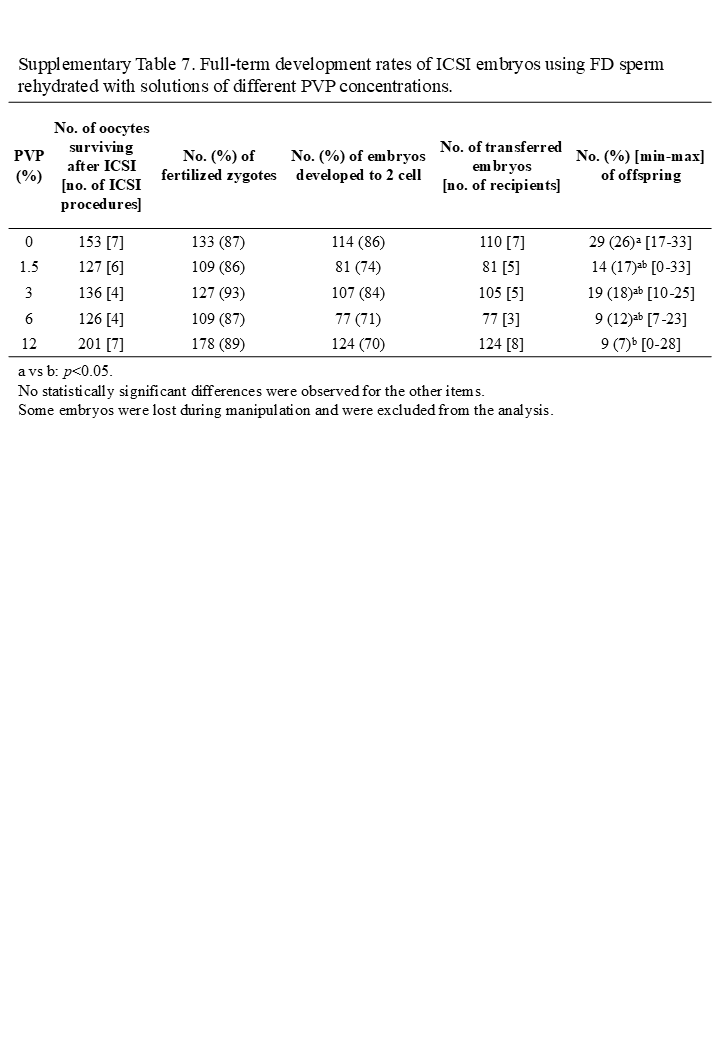

Supplement: S7 Table — (TIF) [file pone.0333682.s007.TIF]

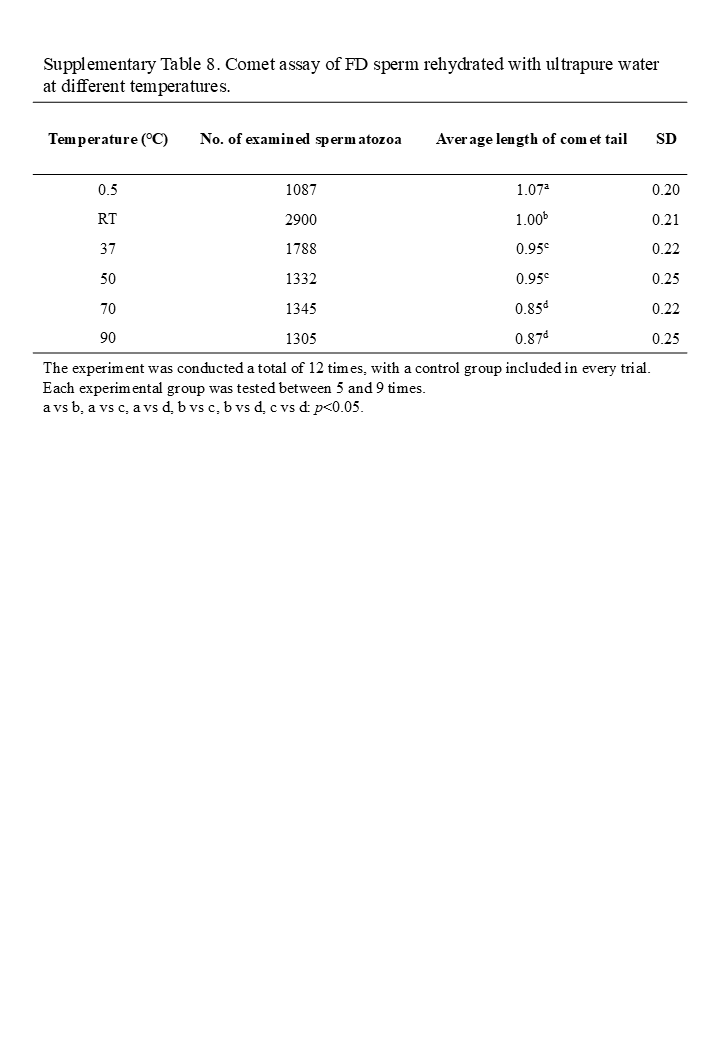

Supplement: S8 Table — (TIF) [file pone.0333682.s008.TIF]

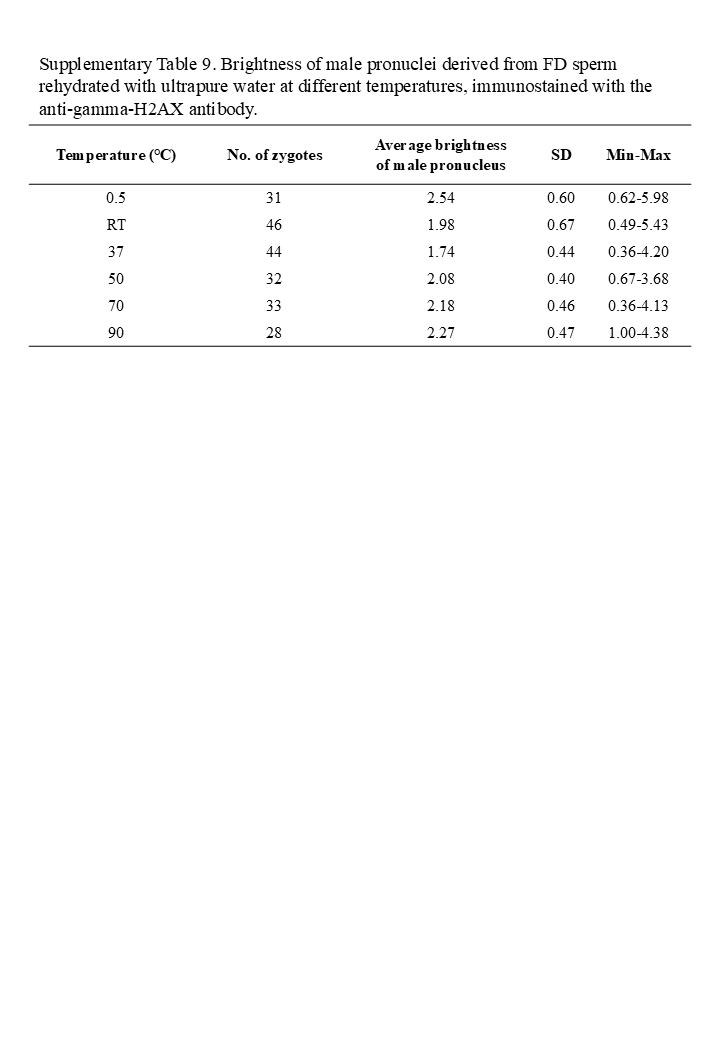

Supplement: S9 Table — (TIF) [file pone.0333682.s009.TIF]

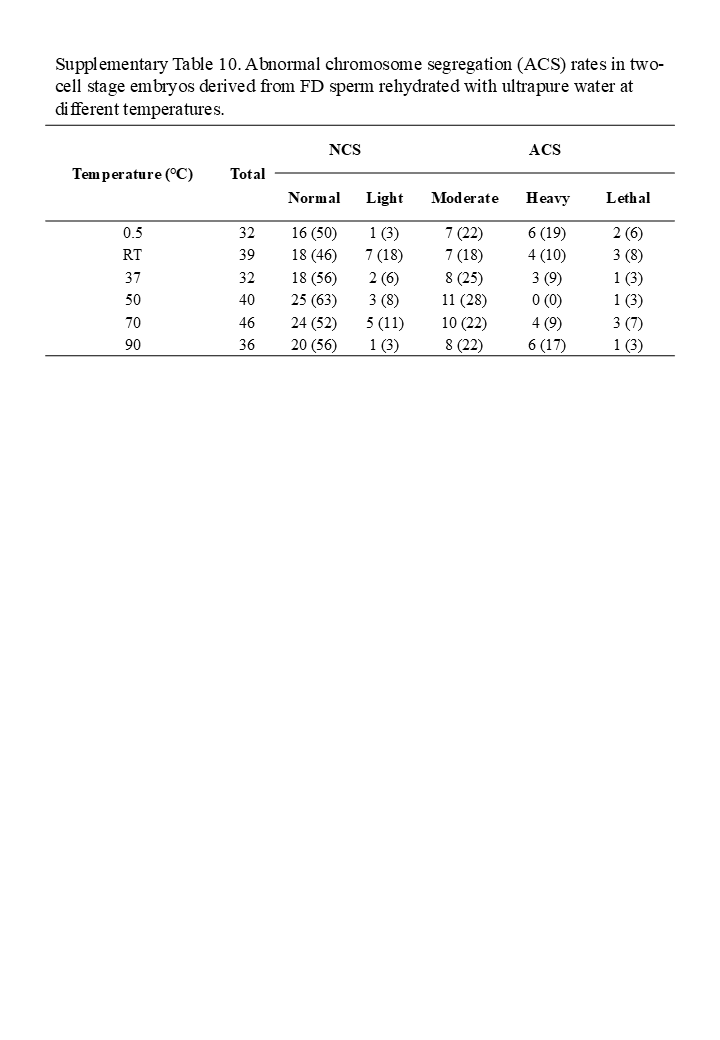

Supplement: S10 Table — (TIF) [file pone.0333682.s010.TIF]
